# Human *Mycobacterium tuberculosis* CD8 T Cell Antigens/Epitopes Identified by a Proteomic Peptide Library

**DOI:** 10.1371/journal.pone.0067016

**Published:** 2013-06-21

**Authors:** David M. Lewinsohn, Gwendolyn M. Swarbrick, Meghan E. Cansler, Megan D. Null, Veena Rajaraman, Marisa M. Frieder, David R. Sherman, Shannon McWeeney, Deborah A. Lewinsohn

**Affiliations:** 1 Division of Pulmonary and Critical Care Medicine, Oregon Health and Sciences University, Portland, Oregon; 2 Portland Veterans Administration Medical Center, Portland, Oregon; 3 Department of Pediatrics, Oregon Health & Sciences University, Portland, Oregon; 4 Oregon Cancer Institute, Oregon Health & Sciences University, Portland, Oregon; 5 Seattle Biomedical Research Institute, Seattle, Washington; Oregon Health and Science University, United States of America

## Abstract

Identification of CD8^+^ T cell antigens/epitopes expressed by human pathogens with large genomes is especially challenging, yet necessary for vaccine development. Immunity to tuberculosis, a leading cause of mortality worldwide, requires CD8^+^ T cell immunity, yet the repertoire of CD8 antigens/epitopes remains undefined. We used integrated computational and proteomic approaches to screen 10% of the *Mycobacterium tuberculosis* (Mtb) proteome for CD8 Mtb antigens. We designed a weighting schema based upon a Multiple Attribute Decision Making:framework to select 10% of the Mtb proteome with a high probability of containing CD8^+^ T cell epitopes. We created a synthetic peptide library consisting of 15-mers overlapping by 11 aa. Using the interferon-γ ELISPOT assay and Mtb-infected dendritic cells as antigen presenting cells, we screened Mtb-specific CD8^+^ T cell clones restricted by classical MHC class I molecules (MHC class Ia molecules), that were isolated from Mtb-infected humans, against this library. Three novel CD8 antigens were unambiguously identified: the EsxJ family (Rv1038c, Rv1197, Rv3620c, Rv2347c, Rv1792), PE9 (Rv1088), and PE_PGRS42 (Rv2487c). The epitopes are B5701-restricted EsxJ_24–34_, B3905-restricted PE9_53–67_, and B3514-restricted PE_PGRS42_48–56_, respectively. The utility of peptide libraries in identifying unknown epitopes recognized by classically restricted CD8^+^ T cells was confirmed, which can be applied to other intracellular pathogens with large size genomes. In addition, we identified three novel Mtb epitopes/antigens that may be evaluated for inclusion in vaccines and/or diagnostics for tuberculosis.

## Introduction

Effective vaccines for many important human pathogens have not yet been developed, and rational design of effective vaccines requires detailed knowledge of correlates of protective immunity. For intracellular pathogens which require T-cell mediated protection such as HIV, CMV, HSV, *Mycobacterium tuberculosis* (Mtb), *Plasmodium* species, *Leishmania* species, *Chlamydiae* species and *Toxoplasma gondii* [1], definition of T cell antigens and epitopes is critical for the definition of these correlates. However, except perhaps for HIV [2], we do not have a comprehensive understanding of human T cell antigens. This is due in part to the large genome size of many human pathogens making production and screening of peptide libraries prohibitively expensive and impractical to perform. Using Mtb as a model pathogen, we have developed an integrated computational and genomic screening approach to identify CD8 antigens.

Tuberculosis (TB), which is caused by infection with Mtb, represents one of the most important causes of morbidity and mortality worldwide, responsible for 9 million cases and 1.7 million deaths each year [3]. Although Bacille Calmette-Guerin (BCG) is the most widely administered vaccine in the world [4], the incidence of TB has remained stable. Thus, elimination of TB worldwide will require a more effective vaccine, and this, in turn, will require a detailed knowledge of protective TB immunity.

Effective T cell responses are essential for TB immunity. Specifically, IFN-γ-producing CD4^+^ Th1 cells have been shown to be critical for protection in the murine TB model [5] and depletion of CD4^+^ T cells in AIDS patients renders them susceptible to TB [6]. Similar to viruses and some other intracellular bacteria such as Salmonella and Listeria [7], a protective role of CD8^+^ T cell responses is supported by the murine TB model [8] and Mtb-specific CD8^+^ T cells are found in Mtb-infected humans and include both T cells classically restricted by HLA-Ia [9]–[12] and non-classically restricted by HLA-E [13] and MR1 [14]. While CD8^+^ T cells have redundant function with CD4^+^ T cells, such as activating infected macrophages with IFN-γ, as well as the use of the granule exocytosis pathway [15], CD8^+^ T cells also have the potential to play a unique role in the recognition and containment of intracellular infection with Mtb. For example, CD8^+^ T cells can lyse infected MHC Class II negative cells such as lung epithelial cells [14]. Furthermore, CD8^+^ T cells preferentially recognize and eliminate heavily infected cells [16]. Thus, CD8^+^ T cells may provide a secondary line of defense when CD4^+^ T cell-activated macrophages fail to contain infection. Furthermore, in the setting of persistent infection with Mtb, CD8^+^ T cells may play a vital role in the immune-surveillance and containment of intracellular infection. The importance of CD8^+^ T cells in the persistent phase of Mtb infection has been highlighted by studies utilizing the mouse [17]–[20] and non-human primate [21] TB models. As a result, vaccines capable of eliciting CD8^+^ T cell responses represent an important goal for vaccine-induced protection from TB.

While CD4 antigens have been extensively characterized [22], [23], the antigens/epitopes recognized by Mtb-specific CD8^+^ T cells remain poorly defined. Previously, most CD8 epitopes had been identified by testing of Mtb peptides selected for high-affinity binding to classical MHC class I molecules (MHC class Ia molecules), mainly HLA-A2 (reviewed in [13]). However, the majority of these epitopes are not strongly recognized by Mtb-infected individuals. In contrast, cases where T cells isolated from Mtb-infected individuals have been used to define epitopes, strongly and commonly recognized epitopes have been identified [10], [11], [24]. In this regard, we have isolated CD8^+^ T cell clones that recognize Mtb-infected dendritic cells (DC) from Mtb-infected individuals and used these T cell clones as a powerful tool to identify novel strongly and commonly recognized CD8 epitopes contained within known CD4 antigens represented by synthetic peptide pools [11]. This was a successful approach in that about two thirds of MHC Ia-restricted Mtb-specific CD8^+^ T cell clones isolated in our laboratory recognize epitopes contained within CD4 antigens. Herein, we screen the CD8^+^ T cell clones for which no antigen was identified by screening against the limited peptide library representing known CD4 antigens, against a synthetic overlapping peptide pool library representing approximately 10% of the Mtb proteome likely to contain CD8 antigens. Using this approach we define three previously unknown CD8 antigens and epitopes processed and presented by Mtb-infected DC and recognized by individuals with latent tuberculosis infection (LTBI) or TB.

## Methods

### Design and synthesis of synthetic peptide library

Initially, a comprehensive evaluation of available genomic and proteomic information was performed. For genomic information, published information was evaluated with regard to its integrity and internal consistency. We first compiled genomic (TubercuList, http://genolist.pasteur.fr/TubercuList/ and [25]) and proteomic (http://web.mpiib-berlin.mpg.de/cgi-bin/pdbs/2d-page/extern/index.cgi and Dr. Karen Dobos, Colorado State University, personal communication) Mtb expression data and meta-data from published sources, from publicly available sources, or available to us as a personal communication. We then loaded these data into an Oracle 9i database.

We designed a weighting schema based upon a Multiple Attribute Decision Making (MADM):framework. First, to calculate the TubercuList Functional Score, each gene was evaluated for weighting based upon functional attributes reflected in TubercuList (Table 1). Functional categories including “PPE/PE”, “cell wall and cell processes”, “virulence, detoxification, adaptation” were given a greater weighting than functional categories such as “intermediary metabolism” due to their potentially greater significance in the immune response to Mtb. For example, cell wall and associated proteins were prioritized because structural components of intracellular pathogens are the first proteins processed and presented to the immune system and often represent CD8 antigens, such as the HIV gag proteins [26], [27]. Finally, because CD4 antigens are preferentially expressed as secreted proteins [23], [28]–[31] and CD4 antigens are often CD8 antigens [11], genes where the word “secreted” was found in the text description were weighted as well. The TubercuList Functional Score was calculated as the total score based on TubercuList attributes. For example a gene classified as a PE/PPE family member (score  = 10) and as in the cell wall (score  = 8) would be assigned a TubercuList Functional Score  = 18. Gene products with a TubercuList Functional Score ≥10 were included in the library. Additionally, gene products where the word “secreted” was identified in the text description, but which nonetheless had a TubercuList Functional Score <10, were also included in the library.

**Table 1:** TubercuList Functional Score.

PE/PPE	10
Cell wall and cell processes	8
Virulence, detoxification, adaptation	7
Lipid metabolism	7
Conserved Hypotheticals	5
Unknown	5
Regulatory proteins	5
All other	−10
“Secreted”	10

Second, a Composite Evidence Score was calculated. This score included additional components of weighting such that each gene was assigned weight based upon three additional lines of evidence (Table 2). These were: 1) Proteomic data: These data included cell-associated proteins from the Erdmann strain, secreted and cell-associated proteins from H37Rv Mtb (http://web.mpiib-berlin.mpg.de/cgi-bin/pdbs/2d-page/extern/index.cgi), and additional data on cell-associated and secreted proteins from the H37Rv strain (Dr. Karen Dobos, Colorado State University, personal communication). As with the TubercuList Functional Score, proteins detected as secreted proteins (supernatant fractions) were given a greater weighting (secreted  = 7) than cell-associated gene products (cell pellet fractions or cell pellet and secreted fractions  = 4). 2) Genomic data: Transcriptional profiling of Mtb gene expression in macrophages [25] was weighted based on constitutive or induced expression. Specifically, gene products with evidence for differential expression associated with adaptation to intracellular growth or stress such that they were induced in one or more experimental condition (1–11 of 12 conditions  = 8) were given greater weighting than genes which were constitutively expressed (12 of 12 conditions  = 6). 3) Not in Bacille Calmeette-Guerin (BCG): Genes not expressed in BCG were assigned a positive weight (score  = 10, Behr: http://www.ncbi.nlm.nih.gov/entrez/query.fcgi?cmd=Retrieve&db=pubmed&dopt=Abstract&list_uids=10348738). To obtain the Composite Evidence Score, the TubercuList Functional Score was added only to additional components scores with non-zero values, and then all three scores were averaged. For example, for Rv3874 (Table S1) Score  = 10, the Composite Evidence Based Score would be (4+8) +0+ (10+8)/3  = 10. Gene products with a Composite Evidence Score ≥5.33 were included in the library.

**Table 2:** Additional components of Composite Evidence Score.

Proteomic	Cellular	4
	Secreted	7
	Both	4
Genomic	12/12 Conditions	6
	1–11 Conditions	8
Not in BCG		10

The synthetic peptide library representing the selected 10% of the Mtb proteome was provided by Jerini Peptide Technologies. The peptide library was comprised of 15-mers overlapping by 11 a.a. (final concentration of individual peptides, 50 nmol) comprising 50 peptide pools in a 96 well format. Construction of the library with 15-mers overlapping by 11 a.a's allowed ex vivo CD8^+^ T cells to be readily detected and ensured that all possible candidate epitopes are represented by at least one 15-mer [11], [32]. Five blank wells and one well of an irrelevant peptide pool representing SIV Gag, were included on each of the nine plates. Peptide pools libraries were stored at −80°C.

### Study participants

Study participants, protocols, and consent forms were approved by the Oregon Health & Science University institutional review board. Written informed consent was obtained from all participants. Individuals with LTBI and uninfected individuals were recruited from employees at Oregon Health &Science University and the general public and active TB donors were recruited from the Multnomah Tuberculosis Clinic, Portland, OR, as previously described [11]. Uninfected individuals were defined as healthy individuals with a history of a negative self-reported tuberculin skin test and no known risk factors for Mtb infection. LTBI was defined as a self-reported history of a tuberculin skin test in a healthy person. Active TB was defined by a person with a recent history of pulmonary TB confirmed by a positive sputum culture. PBMCs were isolated from whole blood obtained by venipuncture or apheresis.

### Media and reagents

For the growth and assay of Mtb-reactive T cell clones, RPMI 1640 supplemented with 10% human sera, gentamicin (50 µg/ml), and 2 mM glutamine (GIBCO BRL, http://www.invitrogen.com/) was used. Mtb strain H37Rv was obtained from the American Type Culture Collection (http://www.atcc.org/) and prepared as previously described [33].

### Cell lines and T cell clones

EBV-transformed B cell lines, lymphoblastoid cell lines (LCL), were either generated in our laboratory using supernatants from the cell line 9B5–8 (American Type Culture Collection) or obtained from the National Marrow Donor Program (http://www.marrow.org/). LCL were maintained by continuous passage as previously described [34]. Mtb-specific T cell clones were isolated from individuals with LTBI or history of active TB, using Mtb-infected DCs as APC and limiting dilution cloning methodology as previously described [11], [33]. Isolated T cell clones specifically recognizing Mtb-specific DC versus uninfected DC by IFN-γ ELISPOT assay, were further characterized. Confirmation of CD8 expression, αβ TCR expression, and determination of the TCR Vβ chain (IOTest Beta Mark Kit, [Beckman Coulter]) were performed by flow cytometry, and determination of HLA-Ia allele restriction was performed by determination of discriminative recognition of single HLA-Ia allele mismatched APC target cells. While our focus was on antigens/epitopes recognized by classically restricted, HLA-Ia-restricted T cell clones, experiments included non-classically restricted, HLA-Ib-restricted T cell clones defined as T cell clones that recognized Mtb-infected autologous DC, Mtb-infected HLA-Ia mismatched DC, and Mtb-infected HLA-Ia mismatched macrophages, but not uninfected autologous DC. For all experiments, T cell clones were expanded using anti-CD3 mAb stimulation as previously described [34], [35].

### Generation and infection of peripheral blood DC

Monocyte-derived DC were prepared according to a modified method of Romani et al. [34], [36]. To generate Mtb-infected DC, day 5 DC (1×10^6^/well) were cultured overnight in the presence of Mtb at a multiplicity of infection  = 30∶1. As heavy infection is required to optimize entry of antigen into the class I processing pathway [16], we have determined that this multiplicity of infection is optimal for detection of Mtb-specific CD8^+^ T cells. After 18 h, the cells were harvested and resuspended in RPMI/10% human serum.

### Screen of peptide library using IFN-γ ELISPOT assay

The IFN-γ ELISPOT assay was performed as described previously [34]. To test the T cell clones against Mtb-infected DC, T cell clones (5000 cells/well) were cultured with autologous DC (20,000 cells/well), which were either uninfected or infected with Mtb, overnight in the IFN-γ ELISPOT assay. For the screen, T cell clones (5,000 cells/well of each clone), autologous DC (20,000 cells/well), IL-2 (0.5 ng/ml) and the peptide pools (5 µg/ml, individual peptides) were tested in the IFN-γ ELISPOT assay using one technical replicate. Negative and positive controls consisted of wells containing T cells and DC either without antigen or without antigen but with inclusion of PHA (10 µg/ml; Sigma Aldrich, http://www.sigmaaldrich.com/), respectively. For all assays, responding T cells were incubated with APCs overnight.

### IFN-γ ELISPOT analysis for fine epitope mapping and HLA-I restricting allele determination

Determination of the minimal epitope recognized and of the HLA-I allele restricting each of the epitopes was performed exactly as described previously [11]. Briefly, to determine the minimal epitope recognized, first the T cell clone (5000 cells/well) was incubated with autologous LCL (20,000 cells/well) in the presence of individual 15-mer peptides (5 µg/ml) as the source of antigen in an IFN-γ ELISPOT assay. Then having identified the unique 15-mer recognized, the IFN-γ ELISPOT assay was repeated using the individual nested peptides (including 8-, 9-, 10-, 11-, and/or 12-mer peptides, 5 g/ml) as a source of antigen. To determine the minimal epitope, T cell clones (1000 cells/well) were incubated with autologous LCL (20,000 cells/well) with the individual nested peptides recognized by the T cell clone over a range of concentrations. By definition, the minimal epitope is the peptide eliciting a T cell response at the lowest concentration. To determine the restricting allele, T cell clones (500 cells/well) were incubated with LCL (20,000 cells/well) that were autologous or expressed HLA alleles matching at only one allele in the presence of peptide representing the epitope (5 µg/ml). A negative control (media, no peptide) and a positive control (PHA, 10 µg/ml) were included in each assay. The restricting allele of each T cell clone was determined by testing against peptide-pulsed LCL sharing at least one HLA allele with the donor.

### Determination of ex vivo T cell frequencies

The ex vivo T cell frequency was also determined as described previously [11]. Briefly, CD8^+^ T cells were selected from PBMCs using magnetic beads resulting in a>97% pure population. Using this CD8 selected population as a source of responding T cells, T cells (5X10^5^, 2.5X10^5^, 1.2X10^5^, and 6X10^4^ cells/well) were incubated with autologous DC (20,000 cells/well) pulsed with the peptide representing the minimal epitope (5 µg/ml) in an IFN-γ ELISPOT assay.

### Data analysis

To determine the ex vivo T cell frequencies, linear regression analysis was used as described previously [11].

## Results

### Creation of peptide library

We utilized integrated computational and proteomic approaches to select 389 genes from the Mtb genome (4011 genes), to comprise the peptide library (Table S1). First, we chose 34 genes of special interest which were comprised of: 1) gene products shown previously in our laboratory to be recognized by CD8^+^ T cells from Mtb-infected individuals (*n* = 10); 2) gene products determined previously to represent CD4 antigens ((22, 23), *n* = 9); and 3) genes that were annotated as “ESAT-like” in Tuberculist (*n* = 15). Next, we selected 256 genes with a Tuberculist Functional Score (assigned as described in Methods) ≥10 and then selected 91 genes with a Composite Evidence-Based Score (assigned as described in Methods) ≥5.33. Finally, we selected genes that were categorized as “secreted” in TubercuList but nonetheless did not have a TubercuList Functional Score ≥10 (*n* = 8) for a total of 389 genes. Secreted proteins were selected based on the observation that secreted proteins from Mtb were often associated with vaccine efficacy [23], [28]–[31]. The thresholds of 5.33 and 10 were chosen to yield a number of remaining genes to attain a total less than 400 genes of interest. The numbers of genes from each Tuberculist functional category are shown in Table 3. Utilizing the finalized gene list, 39,499 peptides were synthesized by Jerini Peptide Technologies to represent the proteins encoded by the 389 selected genes. Each peptide (50 nmol) was synthesized individually and then pooled into 789 pools (50 peptides/pool) in a 96 well format in a total of nine plates.

**Table 3:** Number of genes according to functional category.

Functional Category^1^	Number of genes
PE/PPE	168
Cell wall and cell processes	134
Conserved hypotheticals	30
Virulence, detoxification, adaptation	29
Intermediary metabolism and respiration	10
Lipid metabolism	9
Regulatory proteins	5
Conserved hypotheticals with an orthologue in *M. bovis*	4

1 Functional categories assigned by TubercuList.

### Proof of principle CFP-10

**Figure 1:**
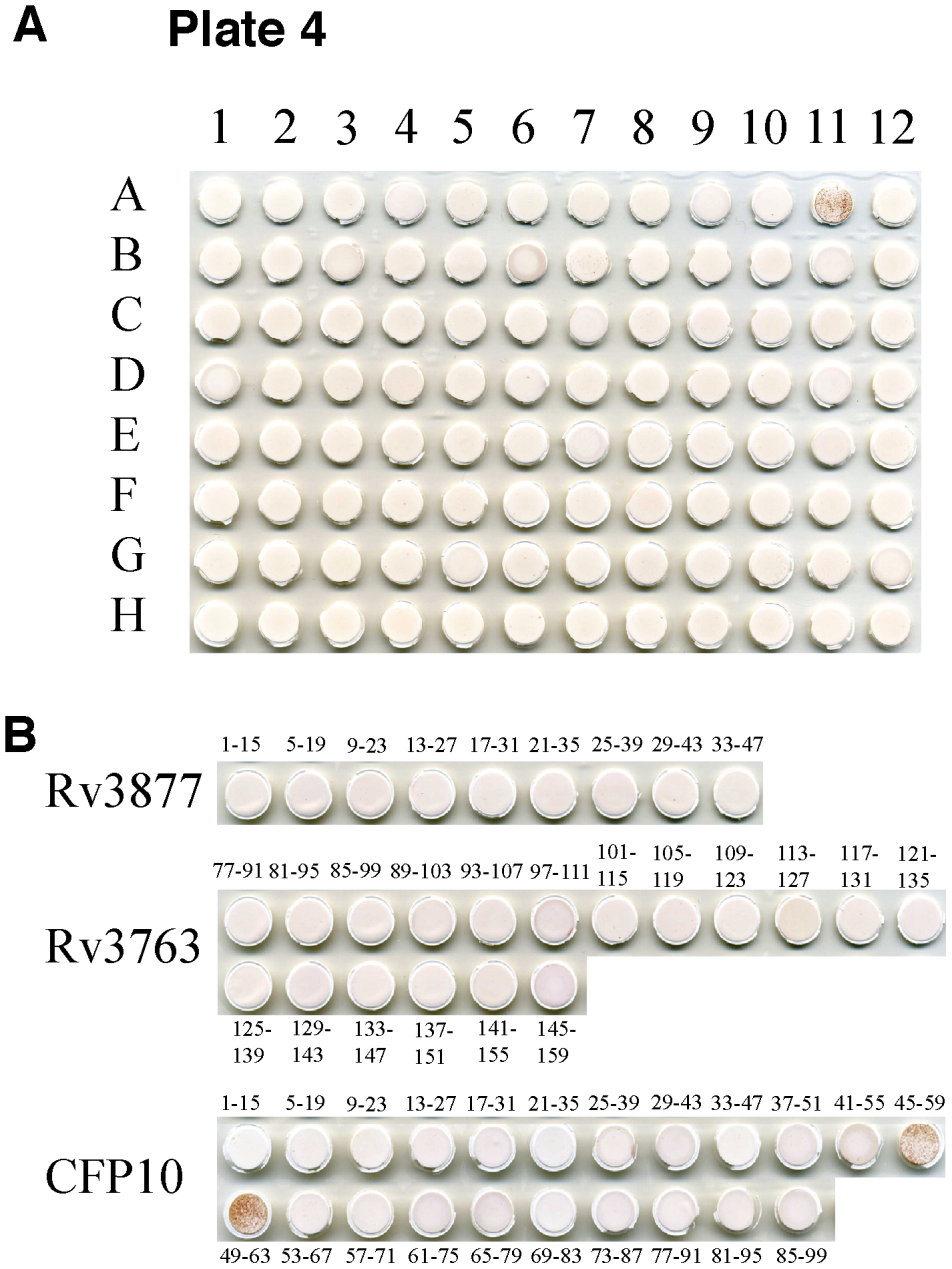
Screen of CFP10-specific CD8^+^ T cell clone, D432 A5, against peptide library. A) D432 A5, D432 D2, D432 E7, D432 E8, D432 H8 and D432 A11 T cells (5000 cells of each clone/well) were incubated with DC (20,000 cells/well) in the presence of the peptide pools (5 µg/ml, individual peptides) and IL-2 (0.5 ng/ml) in single wells in the IFN-γ ELISPOT assay. Positive well is plate 4 well A11. B) To identify the epitope recognized by T cell clone, D432 A5, T cells (5,000 cells/well) were incubated in single wells with autologous LCL (20,000 cells/well) and individual 15 aa peptides from Rv0377, Rv3763 and CFP10 (5 µg/ml) that together constitute the peptide pool from Plate 4 well A11. IFN-γ was assessed by ELISPOT after 18 hours of co-culture. Pictures of ELISPOT wells are shown.

Before screening the T cell clones against the genomic peptide library, the clones were first expanded and tested against Mtb-infected DC to ensure that each clone from this particular expansion yielded a robust Mtb-specific signal in the IFN-γ ELISPOT assay. The expansion of the T cell clone was considered acceptable for use in the peptide library screen if the response of the T cell clone to uninfected DC was <10 spot forming units (SFU)/well and to Mtb-infected DC was >50 SFU/well. To conserve on the use of the genomic peptide library, up to six different T cell clones were pooled and screened in an IFN-γ ELISPOT in the presence of the peptide pools, autologous DC and IL-2. Only one technical replicate was done per pool because, in our experience, 5000 T cell clones per well with a peptide antigen will produce an overwhelmingly positive response, resulting in a clear yes/no answer, while conserving the use of the peptide library. Negative and positive controls were included as described in the methods. As a proof of principle, a CFP-10-specific classically Ia-restricted CD8^+^ T cell clone, D432 A5, was included in the first library screen with 4 classically restricted (D432 D2, D432 E7, D432 E8, D432 H8) and 1 non-classically restricted T cell clone (D432 B12) with unknown antigen specificity. T cells responded to plate 4 of 9, well A11, which contained 22 overlapping peptides representing CFP-10, as well as 9 peptides partially representing Rv3877, and 18 peptides partially representing 19 kd (Figure 1A). T cell clones were then tested against the individual peptides comprising this peptide pool. As expected, D432 A5 recognized two distinct 15-mers CFP-10_45–59_ and CFP-10_49–63_, both of which contain the minimal epitope, CFP-10_49–58_ (Figure 1b).

### Summary of T cell clone screening results

Classically restricted CD8^+^ T cell clones isolated from ten donors (LTBI, *n* = 4; active TB, *n* = 6) had been previously screened against peptide pools representing known CD4 antigens which represented the following proteins: 1) Esat-6, Rv3875; 2) CFP-10, Rv3874; 3) Ag85B, Rv1886c; 4) Mtb9.8, EsxG, Rv0287; 5) 19 kd lipoprotein, Rv3763; 6) TB8.4,Mtb8.4, Rv1174c; 7) Mtb39a, Rv1196; and 8) Mtb9.9, family of ESAT-6 like proteins comprised of EsxN, Rv1793; EsxO, Rv2346c; EsxI, Rv1037c; EsxV, Rv3619c; and EsxL, Rv1198. T cell clones that recognized one of these eight antigens had minimal epitopes mapped as described previously [11]. Mtb-specific, classically restricted T cell clones that did not recognize any of these eight antigens (*n* = 14) isolated from donors with LTBI (D504) or active TB (D432, D466), as well as non-classically restricted T cell clones from these same donors (*n* = 33) were screened against the peptide library. The epitope recognized by 9 of the 14 classically restricted T cell clones was defined. These included 3 of 7 T cell clones isolated from D432, which recognized three distinct epitopes, and all 6 of 6 T cell clones isolated from D504, which all recognized one epitope. Three of four epitopes represent novel epitopes that have not been previously defined. The antigen/epitope recognized by the single T cell clone from D466 was not identified using the peptide library, nor were any of the antigens recognized by non-classically restricted T cell clones.

### Example of epitope identification: T cell clones recognizing EsxJ

In one experiment, five T cell clones from D504, including four classically-restricted T cell clones (D504 B6, D504 B10, D504 F9 and D504 H6) and one non-classically restricted T cell clone (D504 A11), were screened against the peptide library. T cells recognized five distinct wells (four wells on plate 3 and one well on plate 4). Each well of the peptide library contained 50 peptides, which represent one to three genes. All gene products represented by peptides in these five wells belong to the Tuberculist category, “Cell wall and cell processes”. For each of the positive wells of this screen, the peptides contained in these wells and the corresponding gene products are summarized in Table 4. EsxI and EsxO are part of the Mtb9.9 family of genes. As each clone had been previously screened against and did not recognize a peptide pool representing this antigen family, these Mtb9.9 proteins (EsxI and EsxO) were not considered further as candidate antigens. The EsxJ family share 98% homology with each other and differ from one another at only three amino acids (Table 5). As four of the five positive wells from the screen contain peptides from one of four EsxJ family members, we predicted that at least one of the T cell clones screened recognized a single epitope represented in all four wells. We also predicted that this epitope would be located at the carboxyl terminus of the EsxJ, as well F10, plate 3 contained only 9 peptides representing the carboxyl terminus of EsxP_1–43_.

**Table 4:** Description of peptides comprising positive wells.

Plate	Well	Rv	Name	# Peptides in Pool	# Peptides in Protein	Description from Tuberculist
3	A12	Rv1037c	EsxI^1^	2	21	Putative Esat-6 like protein EsxI (Esat-6 like protein 1)
3	A12	Rv1038c	EsxJ^2^	22	22	Esat-6 like protein EsxJ (Esat-6 like protein 2)
3	A12	Rv1072	Rv1072	26	67	Probably conserved transmembrane protein
3	B6	Rv1184c	Rv1184c	20	87	Possible exported protein
3	B6	Rv1197	EsxK^2^	22	22	Esat-6 like protein EsxK (Esat-6 like protein 3)
3	B6	Rv1198	EsxL^1^	8	21	Esat-6 like protein EsxL (Esat-6 like protein 4
3	F10	Rv2330c	LppP	20	41	Probably lipoprotein LPPP
3	F10	Rv2346c	EsxO^1^	21	21	Putative Esat-6 like protein EsxO (Esat-6 like protein 6)
3	F10	Rv2347c	EsxP^2^	9	22	Putative Esat-6 like protein EsxP (Esat-6 like protein 7)
3	G7	Rv2686c	Rv2686c	44	61	Probably antibiotic-transport integral leucine and alanine and valine rich protein ABC transporter
3	G7	Rv2687c	Rv2687c	6	57	Probably antibiotic-transport integral leucine and valine rich protein ABC transporter
4	A8	Rv3620c	EsxW^2^	17	22	Putative Esat-6 like protein EsxW (Esat-6 like protein 10)
4	A8	Rv3641c	Fic	33	50	Possible cell filamentation protein FIC

1 Member of Mtb 9.9 gene family.

2 Member of EsxJ gene family.

**Table 5:** Esx J family members.

EsxJ	MASRFMTDPHAMRDMAGRFEVHAQTVEDEARRMWASAQNISGAGWSGMAEATSLD
EsxW	- TS - - - - - - - - - - - - - - - - - - - - - - - - - - - - - - - - - - - - - - - - - - - - - - - - - - - - - - -
EsxK	- AS - - - - - - - - - - - - - - - - - - - - - - - - - - - - - - - - - - - - - - - - - - - - - - - - - - - - - - -
EsxP	- AT - - - - - - - - - - - - - - - - - - - - - - - - - - - - - - - - - - - - - - - - - - - - - - - - - - - - - - -
EsxJ	TMTQMNQAFRNIVNMLHGVRDGLVRDANNYEQQEQASQQILSS
EsxW	-- --T- - - - - - - - - - - - - - - - - - - - - - - - - - - - - - - - - - - - - - - - - - - - - -
EsxK	-- --A- - - - - - - - - - - - - - - - - - - - - - - - - - - - - - - - - - - - - - - - - - - - - -
EsxP	-- --A- - - - - - - - - - - - - - - - - - - - - - - - - - - - - - - - - - - - - - - - - - - - -

**Figure 2:**
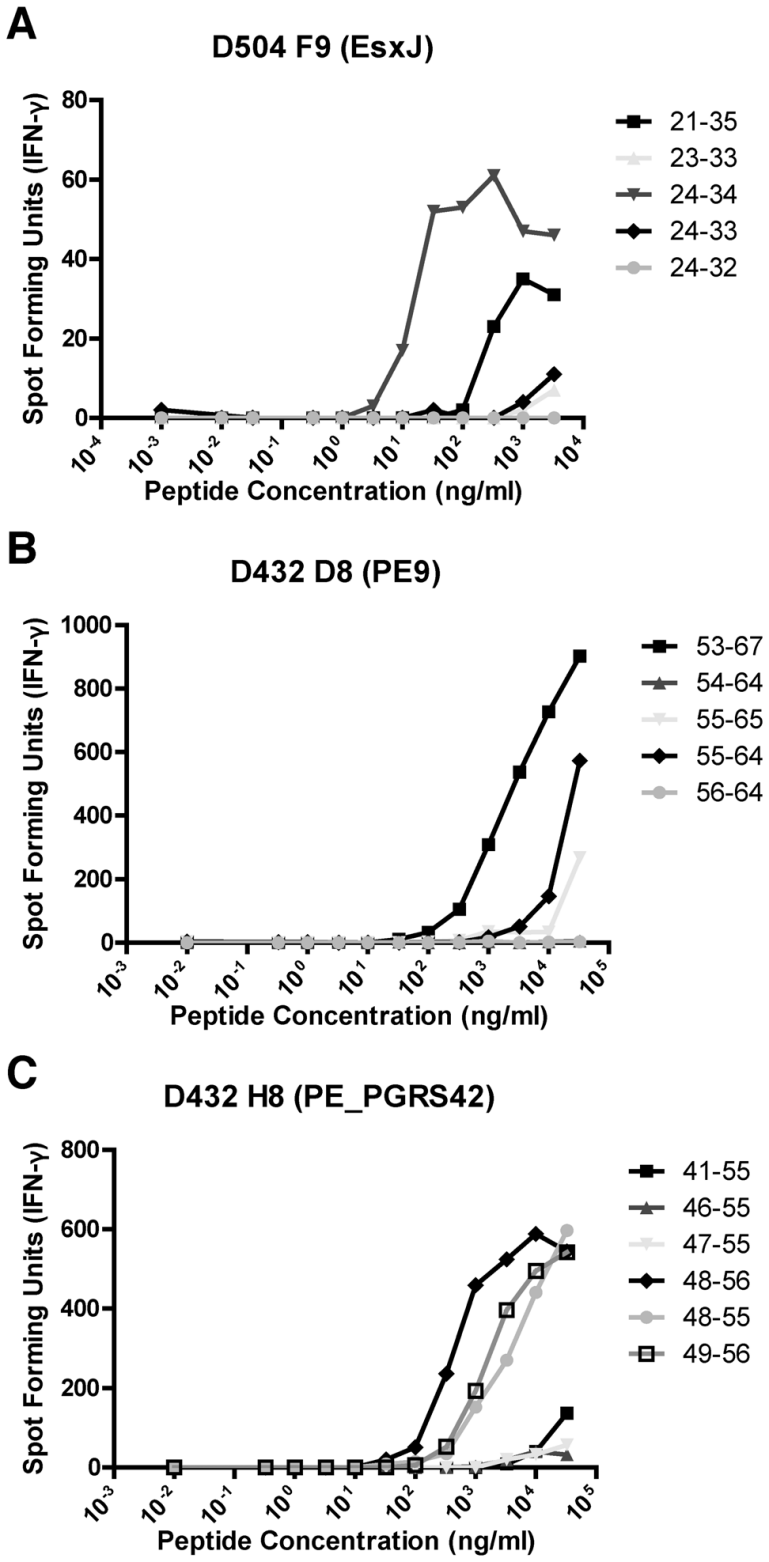
Epitope mapping of EsxJ-specific CD8^+^ T cell clones. To map the minimal epitopes of CD8^+^ T cell clones, A) D504 F9, B) 432 D8, and C) D432 H8, autologous LCL (20,000 cells/well) were pulsed with peptide at the concentrations indicated and co-cultured with T cells (1000 cells/well) in duplicate wells. IFN-γ was assessed by ELISPOT after 18 h co-culture. Each point represents the mean of duplicate determinations.

We next screened each of the T cell clones individually against individual 15-mers representing the EsxJ family, including multiple peptides representing divergent sequences of the family members. Each T cell clone was also screened against individual peptides (*n* = 50) contained in well G7, plate 3, representing gene products of most of Rv2686c and part of Rv2687c. All four classically-restricted T cell clones recognized only one 15-mer, EsxJ_21–35_ which represents a conserved region of the EsxJ family and which was present in all four wells containing peptides representing EsxJ family members. Next, the individual T cell clones were screened against all possible 9-, 10-, and 11-mers contained within EsxJ_21–35_. As shown for one representative clone, D504 F9, (Figure 2A), all four T cell clones recognizing EsxJ_21–35_ also recognized peptide EsxJ_24–34_ at the lowest concentration compared to the other peptides tested, defining EsxJ_24–34_ as the minimal epitope.

### Summary of characterization of T cell clones

In a separate experiment, an additional two T cell clones from D504, D504 D2 and D504 D12, (total, *n* = 6) were screened against the peptide library and also recognized minimal epitope EsxJ_24–34_. The peptide library screen of one T cell clone from donor D466, D466 C7, was negative. Finally, seven T cell clones from D432 were screened. D432 A5 recognizes CFP-10_49–58_ as shown above. D432 D8 recognized a well containing peptides representing PE9. Within PE9, this clone recognized a single 15-mer, PE9_53–67_. D432 D8 was then screened against all possible 9-, 10-, and 11-mers contained in PE9_53–67_, but no nested peptide was recognized at a lower concentration than PE9_53–67_ (Figure 2B). Thus, we were unable to define the minimal epitope recognized by D432 D8 within PE9_53–57_. Finally D432 H8 recognized a well containing peptides representing PE_PGRS42 and a single 15-mer, PE_PGS42_41–55_. D432 H8 recognized PE_PGS42_48–56_ at the lowest concentration compared to the other peptides tested, defining PE_PGS42_48–56_ as the minimal epitope (Figure 2C).

Further characterization of the three T cell clones recognizing novel epitopes is summarized in Table 6. All three clones are restricted by HLA-B alleles. The minimal epitopes recognized by EsxJ-specific D503 F9 and PE_PGRS42-specific D432 H8 are 11 and 9 aa in length, respectively. D432 D8 did not recognize any of all possible 8-, 9-, 10-, 11- and 12-mers within the 15-mer, PE9_53–67_, and therefore, a minimal epitope could not be determined. However, recognition of PE9_53–67_ by D432 D8 was blocked by pan-human MHC Class I blocking antibody, W6/32, confirming the HLA-B restriction of this clone. Furthermore, D432 D8 recognized a second, independently synthesized PE9_53–67_ peptide, suggesting that results with the original synthesized peptide were not due to recognition of a second, contaminating peptide. While the nested peptide approach is generally a productive means by which to identify minimal epitopes, we have been unsuccessful in a small minority (<5%) of epitopes recognized by human HIV-specific and Mtb-specific CD8^+^ T cell clones [11], [24], [37].

**Table 6:** Summary of CD8^+^ T cell epitopes identified.

Donor	Clone^1^	Protein^2^	HLA-Restricting Allele	Epitope Location	Epitope Sequence	Epitope-Specific T cells^3^	V Beta Region
504	F9 (6)	EsxJ	B5701	24–34	QTVEDEARRMW	84	IND
432	D8 (1)	PE9	B3905	53–67	RLFNANAEEYHALSA	94	8
432	H8 (1)	PE_PGRS42	B3514	48–56	SAAIAGLFG	78	7.1

1 Number of sister clones is in parentheses.

2 TubercuList accession numbers are EsxJ (Rv1038c), PE9 (Rv1088), and PE_PGRS42 (2487c).

3 IFN-γ spot forming units per 250,000 CD8^+^ T cells. IND, indeterminate.

To determine if epitopes defined were strongly recognized by CD8^+^ T cells in persons from whom the clones were isolated, we determined ex vivo effector cell frequencies using CD8^+^ T cells isolated from donor PBMCs and autologous DC pulsed with peptide as previously described [11](Table 6). CD8^+^ T cells from D504 recognized EsxJ_24–34_ at a frequency of 84 per 250,000 CD8^+^ T cells. D432 CD8^+^ T cells recognized PE9_53–57_ and PE_PGRS42_48–56_, at frequencies of 94 and 78 per 250,000 CD8^+^ T cells, respectively. Consistent with the strong ex vivo effector cell response to EsxJ_24–34_, multiple T cell clones recognizing this epitope were isolated from D504. We attempted to confirm the clonal relationship between daughter clones by TCR Vβ staining, however, the staining was indeterminate. The flow cytometric method used provides approximately 70% coverage of the normal human TCRVβ, thus this data is suggestive, though not definitive that the six EsxJ-specific D504 CD8^+^ T cell clones represent daughter clones.

## Discussion

We have used a T cell-centered approach to define novel TB antigens and epitopes [10], [11], [24]. Specifically, our first approach was to use T cell clones isolated from Mtb-infected individuals to screen a limited peptide library representing eight known CD4 antigens. Using this approach we were able to identify the antigen/epitopes recognized by many of the classically Ia-restricted Mtb-specific T cells clones isolated in our laboratory. We noted that these epitopes were strongly recognized by CD8^+^ T cells in the persons from whom the T cell clones were isolated and predominantly restricted by HLA-B. Moreover, we noted that the majority of minimal epitopes were longer than nine amino acids. In considering a broader approach to identify the antigen(s) recognized by the remaining T cell clones, we noted that unlike a pathogen with a relatively small genome, such as HIV or hepatitis C virus, creating a peptide library representing the entire Mtb genome would be prohibitive both in terms of cost and labor. Therefore, we developed a weighting schema to select 10% of the Mtb proteome that we predicted would be enriched for CD8 antigens. This library was then screened with CD8^+^ T cell clones with unknown antigen specificity. Using this approach, we identified three new CD8 antigens/epitopes recognized by Mtb-infected T cells that are not known CD4 antigens and hence could not have been detected using our more limited peptide library based upon known CD4 antigens. In addition, all three new CD8 epitopes are restricted by less common HLA-B alleles infrequently interrogated with peptide binding prediction algorithms and hence unlikely to have been revealed with these methods. Finally, strong ex vivo CD8^+^ T cell effector responses were detected similar to our previous observations using a T cell-centered approach [11].

Despite screening the peptide library with over 30 non-classically restricted Mtb-reactive CD8^+^ T cell clones, we were unsuccessful in defining any non-classically restricted antigens. These MR1 [14] and HLA-E [34], restricted CD8^+^ T cell clones recognize protein antigens found within the Mtb cell wall, and we postulate may be either post-translationally modified or non-peptidic. By contrast, using the peptide library representing 10% of the Mtb proteome, in addition to the limited peptide library representing eight known CD4 antigens, we have now identified cognate antigens recognized by 51 of 56 of the HLA-Ia restricted CD8^+^ T cell clones we have isolated. Furthermore, four of five of the clones for which the cognate antigen was not identified were derived from the same donor. Based our prior observations [11], it is likely that these clones recognize less than four distinct antigens/epitopes. Hence, this represents a productive approach for novel discovery of classically restricted CD8 antigens. Identification of the antigen(s) recognized by the non-classically restricted CD8^+^ T cell clones with unknown specificity may require distinct approaches such as an alternative proteomics approach and/or screening an Mtb library expressed in mycobacteria.

The definition of three more CD8 epitopes restricted by HLA-B alleles is consistent with our previous observations [11] and further strengthens the evidence that TB CD8 antigens are preferentially restricted by HLA-B. The reasons for this predominance have not been delineated. However, we speculate that this bias could possibly be due to preferential binding of Mtb-derived peptides to HLA-B, selective upregulation of HLA-B with infection with Mtb, preferential recruitment of HLA-B alleles to the Mtb-containing phagosome [38], and/or selective interference with HLA-A processing and presentation by Mtb. We doubt that our cloning methodology is biased towards isolation of HLA-B- over HLA-A-restricted T cell clones, as using identical methodology we have not observed this skewing for vaccinia-, CMV-, and influenza-specific CD8^+^ T cell clones (D. Lewinsohn, unpublished data and [39]. These data are also consistent with data that suggested that HLA-B-restricted HIV-specific CD8^+^ T cell responses predominated over HLA-A-restricted T cell responses in infected individuals, and were more closely linked with control of HIV viremia [40]. Hence, collective data support the hypothesis that HLA-B-restricted responses may be important for control of both bacterial and viral pathogens.

While the EsxJ family members have not been previously described as either CD4 or CD8 antigens, related ESAT-6, CFP-10, and some other ESAT-6 family members are known human CD4 antigens [41] and ESAT-6 and CFP-10 are known CD8 antigens [11]. All of these are secreted antigens, which in animal models and humans have been shown to be more immunogenic than cell-associated antigens and in animal models are associated with protection [23], [28]–[31]. Our results further demonstrate the immunogenic potential of members of the PE/PPE family of mycobacterial proteins. The PE/PPE proteins are highly variable, unique to mycobacterial species, and comprise 4.2% of the Mtb genome (TubercuList, http://genolist.pasteur.fr/TubercuList/). Unlike ESAT-6 and CFP-10, EsxJ, PE9, and PE_PGRS42 are expressed in BCG (http://www.uniprot.org). Therefore, inclusion of these antigens in recent promising LTBI diagnostics which have increased specificity for Mtb infection based upon measurement of CD4^+^ T cells responses to CFP-10 and ESAT-6 (QuantiFERON, Cellestis Corp and T-spot.TB, Oxford Immunotech), would unlikely increase the performances of these tests. However, these new antigens warrant further investigations as CD8^+^ T cell based TB diagnostics and as candidates in inclusion of TB vaccine candidates. In this regard, we have previously demonstrated that CD8 responses to antigens found in both Mtb and environmental mycobacteria are strongly associated with infection with Mtb [11].

There are several significant infectious causes of morbidity and mortality for which vaccines have yet to be developed, and for which host immunity is critically dependent upon CD4^+^ and/or CD8^+^ T cell immunity [1]. Several of these vaccine targets, including HSV, Mtb, *Plasmodium* sp., *Chlamydia* sp., and *Leishmania* sp. possess relatively large genomes, making whole genome screens of peptide libraries for T cell antigens relatively impractical. This integrated computational and proteomic screening approach for the identification of CD8 antigens could be applied to these other pathogens with large genome size. Moreover, such peptide libraries can be used to define the magnitude and breadth of ex vivo CD8^+^ T effector cell responses. In this regard, current work is underway to screen our peptide library with CD8^+^ T cells from Mtb-infected persons directly ex vivo to define novel CD8 antigens which are strongly and/or commonly recognized.

## Supporting Information

Table S1 **List of genes included in the peptide library.**
(DOC)
