# Supplementary material for: Human Mycobacterium tuberculosis CD8 T Cell Antigens/Epitopes Identified by a Proteomic Peptide Library
Source: PLoS One. 2013 Jun 21;8(6):e67016. doi: 10.1371/journal.pone.0067016 (PMC3689843; doi:10.1371/journal.pone.0067016)
Supplement: Table S1 — List of genes included in the peptide library. (DOC) [file pone.0067016.s001.doc]

| **Table S1. List of genes included in the peptide library^1,2^** | | | | | | | | | |
| --- | --- | --- | --- | --- | --- | --- | --- | --- | --- |
| Rv Number | Tuberculist Functional Score | Proteomic score | Expression score | Not in BCG score | composite evidence based weight | Known CD4/CD8 Antigen | Secreted in Text? | Functional Category | Gene Name |
| Rv0129c | 18 | 22 | 0 | 0 | 7.3333 | **yes** | yes | lipid metabolism | fbpC |
| Rv0287 | 8 | 15 | 0 | 0 | 5 | **yes** | no | Cell Wall | esxG |
| Rv0456c | 8 | 0 | 0 | 0 | 0 | **yes** | no | lipid metabolism | echA2 |
| Rv0915c | 11 | 0 | 0 | 0 | 0 | **yes** | no | PE/PPE | PPE14 |
| Rv0916c | 10 | 0 | 0 | 0 | 0 | **yes** | no | PE/PPE | PE7 |
| Rv0983 | 0 | 7 | 0 | 0 | 2.3333 | **yes** | yes | Other | pepD |
| Rv1174c | 18 | 0 | 0 | 0 | 0 | **yes** | yes | Cell Wall | TB8.4 |
| Rv1196 | 11 | 0 | 0 | 0 | 0 | **yes** | no | PE/PPE | PPE18 |
| Rv1361c | 11 | 0 | 0 | 0 | 0 | **yes** | no | PE/PPE | PPE19 |
| Rv1793 | 9 | 16 | 0 | 0 | 5.3333 | **yes** | no | Cell Wall | esxN |
| Rv1886c | 18 | 22 | 0 | 0 | 7.3333 | **yes** | yes | lipid metabolism | fbpB |
| Rv2376c | 18 | 0 | 0 | 0 | 0 | **yes** | yes | Cell Wall | cfp2 |
| Rv3478 | 11 | 0 | 0 | 0 | 0 | **yes** | no | PE/PPE | PPE60 |
| Rv3763 | 8 | 12 | 0 | 0 | 4 | **yes** | no | Cell Wall | lpqH |
| Rv3803c | 18 | 22 | 0 | 0 | 7.3333 | **yes** | yes | lipid metabolism | fbpD |
| Rv3804c | 18 | 22 | 0 | 0 | 7.3333 | **yes** | yes | lipid metabolism | fbpA |
| Rv3874 | 8 | 12 | 0 | 18 | 10 | **yes** | no | Cell Wall | esxB |
| Rv3875 | 18 | 23 | 0 | 29 | 17.3333 | **yes** | yes | Cell Wall | esxA |
| Rv3914 | 0 | 4 | 0 | 0 | 1.3333 | **yes** | yes | Other | trxC |
| Rv0288 | 9 | 0 | 0 | 0 | 0 | no | no | Cell Wall | **esxH** |
| Rv1037c | 9 | 0 | 0 | 0 | 0 | no | no | Cell Wall | **esxI** |
| Rv1038c | 8 | 12 | 0 | 0 | 4 | no | no | Cell Wall | **esxJ** |
| Rv1197 | 8 | 12 | 0 | 0 | 4 | no | no | Cell Wall | **esxK** |
| Rv1198 | 9 | 16 | 0 | 0 | 5.3333 | no | no | Cell Wall | **esxL** |
| Rv2346c | 9 | 16 | 0 | 0 | 5.3333 | no | no | Cell Wall | **esxO** |
| Rv2347c | 8 | 12 | 0 | 0 | 4 | no | no | Cell Wall | **esxP** |
| Rv3017c | 19 | 0 | 0 | 0 | 0 | no | yes | Cell Wall | **esxQ** |
| Rv3019c | 19 | 0 | 0 | 0 | 0 | no | yes | Cell Wall | **esxR** |
| Rv3444c | 19 | 0 | 0 | 0 | 0 | no | yes | Cell Wall | **esxT** |
| Rv3619c | 9 | 0 | 0 | 0 | 0 | no | no | Cell Wall | **esxV** |
| Rv3620c | 8 | 12 | 0 | 0 | 4 | no | no | Cell Wall | **esxW** |
| Rv3890c | 8 | 0 | 0 | 0 | 0 | no | no | Cell Wall | **esxC** |
| Rv3904c | 8 | 0 | 0 | 0 | 0 | no | no | Cell Wall | **esxE** |
| Rv3905c | 8 | 0 | 0 | 0 | 0 | no | no | Cell Wall | **esxF** |
| Rv0309 | **18** | 0 | 0 | 28 | 9.3333 | no | yes | Cell Wall | Rv0309 |
| Rv2875 | **18** | 0 | 26 | 0 | 8.6667 | no | yes | Cell Wall | mpt70 |
| Rv2873 | **18** | 0 | 26 | 0 | 8.6667 | no | yes | Cell Wall | mpt83 |
| Rv0584 | **18** | 0 | 26 | 0 | 8.6667 | no | yes | Cell Wall | Rv0584 |
| Rv1860 | **18** | 25 | 0 | 0 | 8.3333 | no | yes | Cell Wall | apa |
| Rv2878c | **18** | 25 | 0 | 0 | 8.3333 | no | yes | Cell Wall | mpt53 |
| Rv2721c | **18** | 25 | 0 | 0 | 8.3333 | no | yes | Cell Wall | Rv2721c |
| Rv3036c | **18** | 25 | 0 | 0 | 8.3333 | no | yes | Cell Wall | TB22.2 |
| Rv0559c | **18** | 22 | 0 | 0 | 7.3333 | no | yes | Cell Wall | Rv0559c |
| Rv1269c | **18** | 22 | 0 | 0 | 7.3333 | no | yes | Cell Wall | Rv1269c |
| Rv3004 | **18** | 0 | 0 | 0 | 0 | no | yes | Cell Wall | cfp6 |
| Rv3666c | **18** | 0 | 0 | 0 | 0 | no | yes | Cell Wall | dppA |
| Rv1677 | **18** | 0 | 0 | 0 | 0 | no | yes | Cell Wall | dsbF |
| Rv2903c | **18** | 0 | 0 | 0 | 0 | no | yes | Cell Wall | lepB |
| Rv2518c | **18** | 0 | 0 | 0 | 0 | no | yes | Cell Wall | lppS |
| Rv2905 | **18** | 0 | 0 | 0 | 0 | no | yes | Cell Wall | lppW |
| Rv3298c | **18** | 0 | 0 | 0 | 0 | no | yes | Cell Wall | lpqC |
| Rv3593 | **18** | 0 | 0 | 0 | 0 | no | yes | Cell Wall | lpqF |
| Rv0237 | **18** | 0 | 0 | 0 | 0 | no | yes | Cell Wall | lpqI |
| Rv0671 | **18** | 0 | 0 | 0 | 0 | no | yes | Cell Wall | lpqP |
| Rv0173 | **18** | 0 | 0 | 0 | 0 | no | yes | Cell Wall | lprK |
| Rv0593 | **18** | 0 | 0 | 0 | 0 | no | yes | Cell Wall | lprL |
| Rv1970 | **18** | 0 | 0 | 0 | 0 | no | yes | Cell Wall | lprM |
| Rv3495c | **18** | 0 | 0 | 0 | 0 | no | yes | Cell Wall | lprN |
| Rv0040c | **18** | 0 | 0 | 0 | 0 | no | yes | Cell Wall | mtc28 |
| Rv0867c | **18** | 0 | 0 | 0 | 0 | no | yes | Cell Wall | rpfA |
| Rv2389c | **18** | 0 | 0 | 0 | 0 | no | yes | Cell Wall | rpfD |
| Rv2450c | **18** | 0 | 0 | 0 | 0 | no | yes | Cell Wall | rpfE |
| Rv0178 | **18** | 0 | 0 | 0 | 0 | no | yes | Cell Wall | Rv0178 |
| Rv0192A | **18** | 0 | 0 | 0 | 0 | no | yes | Cell Wall | Rv0192A |
| Rv0236A | **18** | 0 | 0 | 0 | 0 | no | yes | Cell Wall | Rv0236A |
| Rv0383c | **18** | 0 | 0 | 0 | 0 | no | yes | Cell Wall | Rv0383c |
| Rv0394c | **18** | 0 | 0 | 0 | 0 | no | yes | Cell Wall | Rv0394c |
| Rv0398c | **18** | 0 | 0 | 0 | 0 | no | yes | Cell Wall | Rv0398c |
| Rv0412c | **18** | 0 | 0 | 0 | 0 | no | yes | Cell Wall | Rv0412c |
| Rv0477 | **18** | 0 | 0 | 0 | 0 | no | yes | Cell Wall | Rv0477 |
| Rv0479c | **18** | 0 | 0 | 0 | 0 | no | yes | Cell Wall | Rv0479c |
| Rv0870c | **18** | 0 | 0 | 0 | 0 | no | yes | Cell Wall | Rv0870c |
| Rv1271c | **18** | 0 | 0 | 0 | 0 | no | yes | Cell Wall | Rv1271c |
| Rv1291c | **18** | 0 | 0 | 0 | 0 | no | yes | Cell Wall | Rv1291c |
| Rv1312 | **18** | 0 | 0 | 0 | 0 | no | yes | Cell Wall | Rv1312 |
| Rv1435c | **18** | 0 | 0 | 0 | 0 | no | yes | Cell Wall | Rv1435c |
| Rv2253 | **18** | 0 | 0 | 0 | 0 | no | yes | Cell Wall | Rv2253 |
| Rv2700 | **18** | 0 | 0 | 0 | 0 | no | yes | Cell Wall | Rv2700 |
| Rv2969c | **18** | 0 | 0 | 0 | 0 | no | yes | Cell Wall | Rv2969c |
| Rv2972c | **18** | 0 | 0 | 0 | 0 | no | yes | Cell Wall | Rv2972c |
| Rv2980 | **18** | 0 | 0 | 0 | 0 | no | yes | Cell Wall | Rv2980 |
| Rv3163c | **18** | 0 | 0 | 0 | 0 | no | yes | Cell Wall | Rv3163c |
| Rv3194c | **18** | 0 | 0 | 0 | 0 | no | yes | Cell Wall | Rv3194c |
| Rv3312A | **18** | 0 | 0 | 0 | 0 | no | yes | Cell Wall | Rv3312A |
| Rv3605c | **18** | 0 | 0 | 0 | 0 | no | yes | Cell Wall | Rv3605c |
| Rv2588c | **18** | 0 | 0 | 0 | 0 | no | yes | Cell Wall | yajC |
| Rv2416c | **17** | 0 | 25 | 0 | 8.3333 | no | yes | Virulence | eis |
| Rv0591 | **17** | 0 | 25 | 0 | 8.3333 | no | yes | Virulence | mce2C |
| Rv1968 | **17** | 0 | 25 | 0 | 8.3333 | no | yes | Virulence | mce3C |
| Rv1971 | **17** | 0 | 25 | 0 | 8.3333 | no | yes | Virulence | mce3F |
| Rv0169 | **17** | 0 | 0 | 0 | 0 | no | yes | Virulence | mce1A |
| Rv0170 | **17** | 0 | 0 | 0 | 0 | no | yes | Virulence | mce1B |
| Rv0171 | **17** | 0 | 0 | 0 | 0 | no | yes | Virulence | mce1C |
| Rv0172 | **17** | 0 | 0 | 0 | 0 | no | yes | Virulence | mce1D |
| Rv0174 | **17** | 0 | 0 | 0 | 0 | no | yes | Virulence | mce1F |
| Rv0589 | **17** | 0 | 0 | 0 | 0 | no | yes | Virulence | mce2A |
| Rv0590 | **17** | 0 | 0 | 0 | 0 | no | yes | Virulence | mce2B |
| Rv0592 | **17** | 0 | 0 | 0 | 0 | no | yes | Virulence | mce2D |
| Rv0594 | **17** | 0 | 0 | 0 | 0 | no | yes | Virulence | mce2F |
| Rv1966 | **17** | 0 | 0 | 0 | 0 | no | yes | Virulence | mce3A |
| Rv1967 | **17** | 0 | 0 | 0 | 0 | no | yes | Virulence | mce3B |
| Rv1969 | **17** | 0 | 0 | 0 | 0 | no | yes | Virulence | mce3D |
| Rv3499c | **17** | 0 | 0 | 0 | 0 | no | yes | Virulence | mce4A |
| Rv3498c | **17** | 0 | 0 | 0 | 0 | no | yes | Virulence | mce4B |
| Rv3497c | **17** | 0 | 0 | 0 | 0 | no | yes | Virulence | mce4C |
| Rv3496c | **17** | 0 | 0 | 0 | 0 | no | yes | Virulence | mce4D |
| Rv3494c | **17** | 0 | 0 | 0 | 0 | no | yes | Virulence | mce4F |
| Rv0024 | **17** | 0 | 0 | 0 | 0 | no | yes | Virulence | Rv0024 |
| Rv2813 | **15** | 0 | 23 | 0 | 7.6667 | no | yes | conserved hypotheticals | Rv2813 |
| Rv3054c | **15** | 0 | 23 | 0 | 7.6667 | no | yes | conserved hypotheticals | Rv3054c |
| Rv0455c | **15** | 22 | 0 | 0 | 7.3333 | no | yes | conserved hypotheticals | Rv0455c |
| Rv0153c | **15** | 0 | 0 | 0 | 0 | no | yes | Regulatory | ptbB |
| Rv2234 | **15** | 0 | 0 | 0 | 0 | no | yes | Regulatory | ptpA |
| Rv0259c | **15** | 0 | 0 | 0 | 0 | no | yes | conserved hypotheticals | Rv0259c |
| Rv0963c | **15** | 0 | 0 | 0 | 0 | no | yes | conserved hypotheticals | Rv0963c |
| Rv1268c | **15** | 0 | 0 | 0 | 0 | no | yes | conserved hypotheticals | Rv1268c |
| Rv2226 | **15** | 0 | 0 | 0 | 0 | no | yes | conserved hypotheticals | Rv2226 |
| Rv2342 | **15** | 0 | 0 | 0 | 0 | no | yes | conserved hypotheticals | Rv2342 |
| Rv2393 | **15** | 0 | 0 | 0 | 0 | no | yes | conserved hypotheticals | Rv2393 |
| Rv2542 | **15** | 0 | 0 | 0 | 0 | no | yes | conserved hypotheticals | Rv2542 |
| Rv3196 | **15** | 0 | 0 | 0 | 0 | no | yes | conserved hypotheticals | Rv3196 |
| Rv3254 | **15** | 0 | 0 | 0 | 0 | no | yes | conserved hypotheticals | Rv3254 |
| Rv3691 | **15** | 0 | 0 | 0 | 0 | no | yes | conserved hypotheticals | Rv3691 |
| Rv3717 | **15** | 0 | 0 | 0 | 0 | no | yes | conserved hypotheticals | Rv3717 |
| Rv3811 | **15** | 0 | 0 | 0 | 0 | no | yes | conserved hypotheticals | Rv3811 |
| Rv3896c | **15** | 0 | 0 | 0 | 0 | no | yes | conserved hypotheticals | Rv3896c |
| Rv3909 | **15** | 0 | 0 | 0 | 0 | no | yes | conserved hypotheticals | Rv3909 |
| Rv3873 | **11** | 0 | 0 | 21 | 7 | no | no | PE/PPE | PPE68 |
| Rv0096 | **11** | 0 | 19 | 0 | 6.3333 | no | no | PE/PPE | PPE1 |
| Rv0442c | **11** | 0 | 19 | 0 | 6.3333 | no | no | PE/PPE | PPE10 |
| Rv0453 | **11** | 0 | 19 | 0 | 6.3333 | no | no | PE/PPE | PPE11 |
| Rv1168c | **11** | 0 | 19 | 0 | 6.3333 | no | no | PE/PPE | PPE17 |
| Rv1548c | **11** | 0 | 19 | 0 | 6.3333 | no | no | PE/PPE | PPE21 |
| Rv1753c | **11** | 0 | 19 | 0 | 6.3333 | no | no | PE/PPE | PPE24 |
| Rv1801 | **11** | 0 | 19 | 0 | 6.3333 | no | no | PE/PPE | PPE29 |
| Rv1917c | **11** | 0 | 19 | 0 | 6.3333 | no | no | PE/PPE | PPE34 |
| Rv2123 | **11** | 0 | 19 | 0 | 6.3333 | no | no | PE/PPE | PPE37 |
| Rv2430c | **11** | 0 | 19 | 0 | 6.3333 | no | no | PE/PPE | PPE41 |
| Rv3347c | **11** | 0 | 19 | 0 | 6.3333 | no | no | PE/PPE | PPE55 |
| Rv3350c | **11** | 0 | 19 | 0 | 6.3333 | no | no | PE/PPE | PPE56 |
| Rv3425 | **11** | 0 | 19 | 0 | 6.3333 | no | no | PE/PPE | PPE57 |
| Rv3532 | **11** | 0 | 19 | 0 | 6.3333 | no | no | PE/PPE | PPE61 |
| Rv3539 | **11** | 0 | 19 | 0 | 6.3333 | no | no | PE/PPE | PPE63 |
| Rv0355c | **11** | 0 | 19 | 0 | 6.3333 | no | no | PE/PPE | PPE8 |
| Rv0755c | **11** | 0 | 0 | 0 | 0 | no | no | PE/PPE | PPE12 |
| Rv0878c | **11** | 0 | 0 | 0 | 0 | no | no | PE/PPE | PPE13 |
| Rv1039c | **11** | 0 | 0 | 0 | 0 | no | no | PE/PPE | PPE15 |
| Rv1135c | **11** | 0 | 0 | 0 | 0 | no | no | PE/PPE | PPE16 |
| Rv0256c | **11** | 0 | 0 | 0 | 0 | no | no | PE/PPE | PPE2 |
| Rv1387 | **11** | 0 | 0 | 0 | 0 | no | no | PE/PPE | PPE20 |
| Rv1705c | **11** | 0 | 0 | 0 | 0 | no | no | PE/PPE | PPE22 |
| Rv1706c | **11** | 0 | 0 | 0 | 0 | no | no | PE/PPE | PPE23 |
| Rv1787 | **11** | 0 | 0 | 0 | 0 | no | no | PE/PPE | PPE25 |
| Rv1789 | **11** | 0 | 0 | 0 | 0 | no | no | PE/PPE | PPE26 |
| Rv1790 | **11** | 0 | 0 | 0 | 0 | no | no | PE/PPE | PPE27 |
| Rv1800 | **11** | 0 | 0 | 0 | 0 | no | no | PE/PPE | PPE28 |
| Rv0280 | **11** | 0 | 0 | 0 | 0 | no | no | PE/PPE | PPE3 |
| Rv1802 | **11** | 0 | 0 | 0 | 0 | no | no | PE/PPE | PPE30 |
| Rv1807 | **11** | 0 | 0 | 0 | 0 | no | no | PE/PPE | PPE31 |
| Rv1808 | **11** | 0 | 0 | 0 | 0 | no | no | PE/PPE | PPE32 |
| Rv1809 | **11** | 0 | 0 | 0 | 0 | no | no | PE/PPE | PPE33 |
| Rv1918c | **11** | 0 | 0 | 0 | 0 | no | no | PE/PPE | PPE35 |
| Rv2108 | **11** | 0 | 0 | 0 | 0 | no | no | PE/PPE | PPE36 |
| Rv2352c | **11** | 0 | 0 | 0 | 0 | no | no | PE/PPE | PPE38 |
| Rv2353c | **11** | 0 | 0 | 0 | 0 | no | no | PE/PPE | PPE39 |
| Rv0286 | **11** | 0 | 0 | 0 | 0 | no | no | PE/PPE | PPE4 |
| Rv2356c | **11** | 0 | 0 | 0 | 0 | no | no | PE/PPE | PPE40 |
| Rv2608 | **11** | 0 | 0 | 0 | 0 | no | no | PE/PPE | PPE42 |
| Rv2768c | **11** | 0 | 0 | 0 | 0 | no | no | PE/PPE | PPE43 |
| Rv2770c | **11** | 0 | 0 | 0 | 0 | no | no | PE/PPE | PPE44 |
| Rv2892c | **11** | 0 | 0 | 0 | 0 | no | no | PE/PPE | PPE45 |
| Rv3018c | **11** | 0 | 0 | 0 | 0 | no | no | PE/PPE | PPE46 |
| Rv3021c | **11** | 0 | 0 | 0 | 0 | no | no | PE/PPE | PPE47 |
| Rv3022c | **11** | 0 | 0 | 0 | 0 | no | no | PE/PPE | PPE48 |
| Rv3125c | **11** | 0 | 0 | 0 | 0 | no | no | PE/PPE | PPE49 |
| Rv0304c | **11** | 0 | 0 | 0 | 0 | no | no | PE/PPE | PPE5 |
| Rv3135 | **11** | 0 | 0 | 0 | 0 | no | no | PE/PPE | PPE50 |
| Rv3136 | **11** | 0 | 0 | 0 | 0 | no | no | PE/PPE | PPE51 |
| Rv3144c | **11** | 0 | 0 | 0 | 0 | no | no | PE/PPE | PPE52 |
| Rv3159c | **11** | 0 | 0 | 0 | 0 | no | no | PE/PPE | PPE53 |
| Rv3343c | **11** | 0 | 0 | 0 | 0 | no | no | PE/PPE | PPE54 |
| Rv3426 | **11** | 0 | 0 | 0 | 0 | no | no | PE/PPE | PPE58 |
| Rv3429 | **11** | 0 | 0 | 0 | 0 | no | no | PE/PPE | PPE59 |
| Rv0305c | **11** | 0 | 0 | 0 | 0 | no | no | PE/PPE | PPE6 |
| Rv3533c | **11** | 0 | 0 | 0 | 0 | no | no | PE/PPE | PPE62 |
| Rv3558 | **11** | 0 | 0 | 0 | 0 | no | no | PE/PPE | PPE64 |
| Rv3621c | **11** | 0 | 0 | 0 | 0 | no | no | PE/PPE | PPE65 |
| Rv3738c | **11** | 0 | 0 | 0 | 0 | no | no | PE/PPE | PPE66 |
| Rv3739c | **11** | 0 | 0 | 0 | 0 | no | no | PE/PPE | PPE67 |
| Rv0388c | **11** | 0 | 0 | 0 | 0 | no | no | PE/PPE | PPE9 |
| Rv1768 | **10** | 0 | 0 | 20 | 6.6667 | no | no | PE/PPE | PE_PGRS31 |
| Rv1983 | **10** | 0 | 0 | 20 | 6.6667 | no | no | PE/PPE | PE_PGRS35 |
| Rv3872 | **10** | 0 | 0 | 20 | 6.6667 | no | no | PE/PPE | PE35 |
| Rv0754 | **10** | 0 | 18 | 0 | 6 | no | no | PE/PPE | PE_PGRS11 |
| Rv1169c | **10** | 0 | 18 | 0 | 6 | no | no | PE/PPE | PE11 |
| Rv2431c | **10** | 0 | 18 | 0 | 6 | no | no | PE/PPE | PE25 |
| Rv3746c | **10** | 0 | 18 | 0 | 6 | no | no | PE/PPE | PE34 |
| Rv0160c | **10** | 0 | 18 | 0 | 6 | no | no | PE/PPE | PE4 |
| Rv0109 | **10** | 0 | 0 | 0 | 0 | no | no | PE/PPE | PE_PGRS1 |
| Rv0747 | **10** | 0 | 0 | 0 | 0 | no | no | PE/PPE | PE_PGRS10 |
| Rv0832 | **10** | 0 | 0 | 0 | 0 | no | no | PE/PPE | PE_PGRS12 |
| Rv0833 | **10** | 0 | 0 | 0 | 0 | no | no | PE/PPE | PE_PGRS13 |
| Rv0834c | **10** | 0 | 0 | 0 | 0 | no | no | PE/PPE | PE_PGRS14 |
| Rv0872c | **10** | 0 | 0 | 0 | 0 | no | no | PE/PPE | PE_PGRS15 |
| Rv0977 | **10** | 0 | 0 | 0 | 0 | no | no | PE/PPE | PE_PGRS16 |
| Rv0978c | **10** | 0 | 0 | 0 | 0 | no | no | PE/PPE | PE_PGRS17 |
| Rv0980c | **10** | 0 | 0 | 0 | 0 | no | no | PE/PPE | PE_PGRS18 |
| Rv1067c | **10** | 0 | 0 | 0 | 0 | no | no | PE/PPE | PE_PGRS19 |
| Rv0124 | **10** | 0 | 0 | 0 | 0 | no | no | PE/PPE | PE_PGRS2 |
| Rv1068c | **10** | 0 | 0 | 0 | 0 | no | no | PE/PPE | PE_PGRS20 |
| Rv1087 | **10** | 0 | 0 | 0 | 0 | no | no | PE/PPE | PE_PGRS21 |
| Rv1091 | **10** | 0 | 0 | 0 | 0 | no | no | PE/PPE | PE_PGRS22 |
| Rv1243c | **10** | 0 | 0 | 0 | 0 | no | no | PE/PPE | PE_PGRS23 |
| Rv1325c | **10** | 0 | 0 | 0 | 0 | no | no | PE/PPE | PE_PGRS24 |
| Rv1396c | **10** | 0 | 0 | 0 | 0 | no | no | PE/PPE | PE_PGRS25 |
| Rv1441c | **10** | 0 | 0 | 0 | 0 | no | no | PE/PPE | PE_PGRS26 |
| Rv1450c | **10** | 0 | 0 | 0 | 0 | no | no | PE/PPE | PE_PGRS27 |
| Rv1452c | **10** | 0 | 0 | 0 | 0 | no | no | PE/PPE | PE_PGRS28 |
| Rv1468c | **10** | 0 | 0 | 0 | 0 | no | no | PE/PPE | PE_PGRS29 |
| Rv0278c | **10** | 0 | 0 | 0 | 0 | no | no | PE/PPE | PE_PGRS3 |
| Rv1651c | **10** | 0 | 0 | 0 | 0 | no | no | PE/PPE | PE_PGRS30 |
| Rv1803c | **10** | 0 | 0 | 0 | 0 | no | no | PE/PPE | PE_PGRS32 |
| Rv1818c | **10** | 0 | 0 | 0 | 0 | no | no | PE/PPE | PE_PGRS33 |
| Rv1840c | **10** | 0 | 0 | 0 | 0 | no | no | PE/PPE | PE_PGRS34 |
| Rv2098c | **10** | 0 | 0 | 0 | 0 | no | no | PE/PPE | PE_PGRS36 |
| Rv2126c | **10** | 0 | 0 | 0 | 0 | no | no | PE/PPE | PE_PGRS37 |
| Rv2162c | **10** | 0 | 0 | 0 | 0 | no | no | PE/PPE | PE_PGRS38 |
| Rv2340c | **10** | 0 | 0 | 0 | 0 | no | no | PE/PPE | PE_PGRS39 |
| Rv0279c | **10** | 0 | 0 | 0 | 0 | no | no | PE/PPE | PE_PGRS4 |
| Rv2371 | **10** | 0 | 0 | 0 | 0 | no | no | PE/PPE | PE_PGRS40 |
| Rv2396 | **10** | 0 | 0 | 0 | 0 | no | no | PE/PPE | PE_PGRS41 |
| Rv2487c | **10** | 0 | 0 | 0 | 0 | no | no | PE/PPE | PE_PGRS42 |
| Rv2490c | **10** | 0 | 0 | 0 | 0 | no | no | PE/PPE | PE_PGRS43 |
| Rv2591 | **10** | 0 | 0 | 0 | 0 | no | no | PE/PPE | PE_PGRS44 |
| Rv2615c | **10** | 0 | 0 | 0 | 0 | no | no | PE/PPE | PE_PGRS45 |
| Rv2634c | **10** | 0 | 0 | 0 | 0 | no | no | PE/PPE | PE_PGRS46 |
| Rv2741 | **10** | 0 | 0 | 0 | 0 | no | no | PE/PPE | PE_PGRS47 |
| Rv2853 | **10** | 0 | 0 | 0 | 0 | no | no | PE/PPE | PE_PGRS48 |
| Rv3344c | **10** | 0 | 0 | 0 | 0 | no | no | PE/PPE | PE_PGRS49 |
| Rv0297 | **10** | 0 | 0 | 0 | 0 | no | no | PE/PPE | PE_PGRS5 |
| Rv3345c | **10** | 0 | 0 | 0 | 0 | no | no | PE/PPE | PE_PGRS50 |
| Rv3367 | **10** | 0 | 0 | 0 | 0 | no | no | PE/PPE | PE_PGRS51 |
| Rv3388 | **10** | 0 | 0 | 0 | 0 | no | no | PE/PPE | PE_PGRS52 |
| Rv3507 | **10** | 0 | 0 | 0 | 0 | no | no | PE/PPE | PE_PGRS53 |
| Rv3508 | **10** | 0 | 0 | 0 | 0 | no | no | PE/PPE | PE_PGRS54 |
| Rv3511 | **10** | 0 | 0 | 0 | 0 | no | no | PE/PPE | PE_PGRS55 |
| Rv3512 | **10** | 0 | 0 | 0 | 0 | no | no | PE/PPE | PE_PGRS56 |
| Rv3514 | **10** | 0 | 0 | 0 | 0 | no | no | PE/PPE | PE_PGRS57 |
| Rv3590c | **10** | 0 | 0 | 0 | 0 | no | no | PE/PPE | PE_PGRS58 |
| Rv3595c | **10** | 0 | 0 | 0 | 0 | no | no | PE/PPE | PE_PGRS59 |
| Rv0532 | **10** | 0 | 0 | 0 | 0 | no | no | PE/PPE | PE_PGRS6 |
| Rv3652 | **10** | 0 | 0 | 0 | 0 | no | no | PE/PPE | PE_PGRS60 |
| Rv3653 | **10** | 0 | 0 | 0 | 0 | no | no | PE/PPE | PE_PGRS61 |
| Rv3812 | **10** | 0 | 0 | 0 | 0 | no | no | PE/PPE | PE_PGRS62 |
| Rv3097c | **10** | 0 | 0 | 0 | 0 | no | no | PE/PPE | PE_PGRS63 |
| Rv0578c | **10** | 0 | 0 | 0 | 0 | no | no | PE/PPE | PE_PGRS7 |
| Rv0742 | **10** | 0 | 0 | 0 | 0 | no | no | PE/PPE | PE_PGRS8 |
| Rv0746 | **10** | 0 | 0 | 0 | 0 | no | no | PE/PPE | PE_PGRS9 |
| Rv0151c | **10** | 0 | 0 | 0 | 0 | no | no | PE/PPE | PE1 |
| Rv1089 | **10** | 0 | 0 | 0 | 0 | no | no | PE/PPE | PE10 |
| Rv1172c | **10** | 0 | 0 | 0 | 0 | no | no | PE/PPE | PE12 |
| Rv1195 | **10** | 0 | 0 | 0 | 0 | no | no | PE/PPE | PE13 |
| Rv1214c | **10** | 0 | 0 | 0 | 0 | no | no | PE/PPE | PE14 |
| Rv1386 | **10** | 0 | 0 | 0 | 0 | no | no | PE/PPE | PE15 |
| Rv1430 | **10** | 0 | 0 | 0 | 0 | no | no | PE/PPE | PE16 |
| Rv1646 | **10** | 0 | 0 | 0 | 0 | no | no | PE/PPE | PE17 |
| Rv1788 | **10** | 0 | 0 | 0 | 0 | no | no | PE/PPE | PE18 |
| Rv1791 | **10** | 0 | 0 | 0 | 0 | no | no | PE/PPE | PE19 |
| Rv0152c | **10** | 0 | 0 | 0 | 0 | no | no | PE/PPE | PE2 |
| Rv1806 | **10** | 0 | 0 | 0 | 0 | no | no | PE/PPE | PE20 |
| Rv2099c | **10** | 0 | 0 | 0 | 0 | no | no | PE/PPE | PE21 |
| Rv2107 | **10** | 0 | 0 | 0 | 0 | no | no | PE/PPE | PE22 |
| Rv2328 | **10** | 0 | 0 | 0 | 0 | no | no | PE/PPE | PE23 |
| Rv2408 | **10** | 0 | 0 | 0 | 0 | no | no | PE/PPE | PE24 |
| Rv2519 | **10** | 0 | 0 | 0 | 0 | no | no | PE/PPE | PE26 |
| Rv2769c | **10** | 0 | 0 | 0 | 0 | no | no | PE/PPE | PE27 |
| Rv3018A | **10** | 0 | 0 | 0 | 0 | no | no | PE/PPE | PE27A |
| Rv3022A | **10** | 0 | 0 | 0 | 0 | no | no | PE/PPE | PE29 |
| Rv0159c | **10** | 0 | 0 | 0 | 0 | no | no | PE/PPE | PE3 |
| Rv3477 | **10** | 0 | 0 | 0 | 0 | no | no | PE/PPE | PE31 |
| Rv3622c | **10** | 0 | 0 | 0 | 0 | no | no | PE/PPE | PE32 |
| Rv3650 | **10** | 0 | 0 | 0 | 0 | no | no | PE/PPE | PE33 |
| Rv3893c | **10** | 0 | 0 | 0 | 0 | no | no | PE/PPE | PE36 |
| Rv0285 | **10** | 0 | 0 | 0 | 0 | no | no | PE/PPE | PE5 |
| Rv0335c | **10** | 0 | 0 | 0 | 0 | no | no | PE/PPE | PE6 |
| Rv1040c | **10** | 0 | 0 | 0 | 0 | no | no | PE/PPE | PE8 |
| Rv1088 | **10** | 0 | 0 | 0 | 0 | no | no | PE/PPE | PE9 |
| Rv3892c | **10** | 0 | 0 | 0 | 0 | no | no | PE/PPE | PPE69 |
| Rv0354c | **10** | 0 | 0 | 0 | 0 | no | no | PE/PPE | PPE7 |
| Rv1759c | **10** | 0 | 0 | 0 | 0 | no | no | PE/PPE | wag22 |
| Rv1986 | 8 | 0 | 16 | 18 | **11.3333** | no | no | Cell Wall | Rv1986 |
| Rv3402c | 8 | 0 | 16 | 18 | **11.3333** | no | no | Cell Wall | Rv3402c |
| Rv1984c | 8 | 15 | 0 | 18 | **11** | no | no | Cell Wall | cfp21 |
| Rv1980c | 8 | 12 | 0 | 18 | **10** | no | no | Cell Wall | mpt64 |
| Rv0824c | 7 | 14 | 15 | 0 | **9.6667** | no | no | lipid metabolism | desA1 |
| Rv0468 | 8 | 12 | 16 | 0 | **9.3333** | no | no | lipid metabolism | fadB2 |
| Rv3403c | 5 | 0 | 13 | 15 | **9.3333** | no | no | conserved hypotheticals | Rv3403c |
| Rv2428 | 7 | 11 | 15 | 0 | **8.6667** | no | no | Virulence | ahpC |
| Rv2429 | 7 | 11 | 15 | 0 | **8.6667** | no | no | Virulence | ahpD |
| Rv0384c | 7 | 11 | 15 | 0 | **8.6667** | no | no | Virulence | clpB |
| Rv0350 | 7 | 11 | 15 | 0 | **8.6667** | no | no | Virulence | dnaK |
| Rv0351 | 7 | 11 | 15 | 0 | **8.6667** | no | no | Virulence | grpE |
| Rv2031c | 7 | 11 | 15 | 0 | **8.6667** | no | no | Virulence | hspX |
| Rv2711 | 5 | 12 | 13 | 0 | **8.3333** | no | no | Regulatory | ideR |
| Rv2015c | 5 | 12 | 13 | 0 | **8.3333** | no | no | conserved hypotheticals | Rv2015c |
| Rv2660c | 5 | 12 | 13 | 0 | **8.3333** | no | no | conserved hypotheticals | Rv2660c |
| Rv0251c | 7 | 11 | 13 | 0 | **8** | no | no | Virulence | hsp |
| Rv1978 | 5 | 9 | 0 | 15 | **8** | no | no | conserved hypotheticals | Rv1978 |
| Rv2744c | 5 | 9 | 13 | 0 | **7.3333** | no | no | conserved hypotheticals | 35kd_ag |
| Rv3133c | 5 | 9 | 13 | 0 | **7.3333** | no | no | Regulatory | devR |
| Rv0080 | 5 | 9 | 13 | 0 | **7.3333** | no | no | conserved hypotheticals | Rv0080 |
| Rv0250c | 5 | 9 | 13 | 0 | **7.3333** | no | no | conserved hypotheticals | Rv0250c |
| Rv0569 | 5 | 9 | 13 | 0 | **7.3333** | no | no | conserved hypotheticals | Rv0569 |
| Rv1404 | 5 | 9 | 13 | 0 | **7.3333** | no | no | Regulatory | Rv1404 |
| Rv1738 | 5 | 9 | 13 | 0 | **7.3333** | no | no | conserved hypotheticals | Rv1738 |
| Rv1996 | 5 | 9 | 13 | 0 | **7.3333** | no | no | conserved hypotheticals | Rv1996 |
| Rv2005c | 5 | 9 | 13 | 0 | **7.3333** | no | no | conserved hypotheticals | Rv2005c |
| Rv2557 | 5 | 9 | 13 | 0 | **7.3333** | no | no | conserved hypotheticals | Rv2557 |
| Rv2619c | 5 | 9 | 13 | 0 | **7.3333** | no | no | conserved hypotheticals | Rv2619c |
| Rv2626c | 5 | 9 | 13 | 0 | **7.3333** | no | no | conserved hypotheticals | Rv2626c |
| Rv3127 | 5 | 9 | 13 | 0 | **7.3333** | no | no | conserved hypotheticals | Rv3127 |
| Rv3555c | 5 | 9 | 13 | 0 | **7.3333** | no | no | conserved hypotheticals | Rv3555c |
| Rv2623 | 5 | 9 | 13 | 0 | **7.3333** | no | no | conserved hypotheticals | TB31.7 |
| Rv1979c | 8 | 0 | 0 | 18 | **6** | no | no | Cell Wall | Rv1979c |
| Rv1987 | 8 | 0 | 0 | 18 | **6** | no | no | Cell Wall | Rv1987 |
| Rv3877 | 8 | 0 | 0 | 18 | **6** | no | no | Cell Wall | Rv3877 |
| Rv2643 | 8 | 0 | 16 | 0 | **5.3333** | no | no | Cell Wall | arsC |
| Rv0917 | 8 | 0 | 16 | 0 | **5.3333** | no | no | Cell Wall | betP |
| Rv1997 | 8 | 0 | 16 | 0 | **5.3333** | no | no | Cell Wall | ctpF |
| Rv1992c | 8 | 0 | 16 | 0 | **5.3333** | no | no | Cell Wall | ctpG |
| Rv3664c | 8 | 0 | 16 | 0 | **5.3333** | no | no | Cell Wall | dppC |
| Rv3516 | 8 | 0 | 16 | 0 | **5.3333** | no | no | lipid metabolism | echA19 |
| Rv1715 | 8 | 0 | 16 | 0 | **5.3333** | no | no | lipid metabolism | fadB3 |
| Rv3641c | 8 | 0 | 16 | 0 | **5.3333** | no | no | Cell Wall | fic |
| Rv2544 | 8 | 0 | 16 | 0 | **5.3333** | no | no | Cell Wall | lppB |
| Rv2270 | 8 | 0 | 16 | 0 | **5.3333** | no | no | Cell Wall | lppN |
| Rv2330c | 8 | 0 | 16 | 0 | **5.3333** | no | no | Cell Wall | lppP |
| Rv1343c | 8 | 0 | 16 | 0 | **5.3333** | no | no | Cell Wall | lprD |
| Rv1737c | 8 | 0 | 16 | 0 | **5.3333** | no | no | Cell Wall | narK2 |
| Rv0102 | 8 | 0 | 16 | 0 | **5.3333** | no | no | Cell Wall | Rv0102 |
| Rv0188 | 8 | 0 | 16 | 0 | **5.3333** | no | no | Cell Wall | Rv0188 |
| Rv0283 | 8 | 0 | 16 | 0 | **5.3333** | no | no | Cell Wall | Rv0283 |
| Rv0284 | 8 | 0 | 16 | 0 | **5.3333** | no | no | Cell Wall | Rv0284 |
| Rv0446c | 8 | 0 | 16 | 0 | **5.3333** | no | no | Cell Wall | Rv0446c |
| Rv0473 | 8 | 0 | 16 | 0 | **5.3333** | no | no | Cell Wall | Rv0473 |
| Rv1072 | 8 | 0 | 16 | 0 | **5.3333** | no | no | Cell Wall | Rv1072 |
| Rv1132 | 8 | 0 | 16 | 0 | **5.3333** | no | no | Cell Wall | Rv1132 |
| Rv1184c | 8 | 0 | 16 | 0 | **5.3333** | no | no | Cell Wall | Rv1184c |
| Rv1258c | 8 | 0 | 16 | 0 | **5.3333** | no | no | Cell Wall | Rv1258c |
| Rv1273c | 8 | 0 | 16 | 0 | **5.3333** | no | no | Cell Wall | Rv1273c |
| Rv1342c | 8 | 0 | 16 | 0 | **5.3333** | no | no | Cell Wall | Rv1342c |
| Rv1348 | 8 | 0 | 16 | 0 | **5.3333** | no | no | Cell Wall | Rv1348 |
| Rv1349 | 8 | 0 | 16 | 0 | **5.3333** | no | no | Cell Wall | Rv1349 |
| Rv1686c | 8 | 0 | 16 | 0 | **5.3333** | no | no | Cell Wall | Rv1686c |
| Rv1687c | 8 | 0 | 16 | 0 | **5.3333** | no | no | Cell Wall | Rv1687c |
| Rv1730c | 8 | 0 | 16 | 0 | **5.3333** | no | no | Cell Wall | Rv1730c |
| Rv1733c | 8 | 0 | 16 | 0 | **5.3333** | no | no | Cell Wall | Rv1733c |
| Rv1735c | 8 | 0 | 16 | 0 | **5.3333** | no | no | Cell Wall | Rv1735c |
| Rv1739c | 8 | 0 | 16 | 0 | **5.3333** | no | no | Cell Wall | Rv1739c |
| Rv1999c | 8 | 0 | 16 | 0 | **5.3333** | no | no | Cell Wall | Rv1999c |
| Rv2025c | 8 | 0 | 16 | 0 | **5.3333** | no | no | Cell Wall | Rv2025c |
| Rv2040c | 8 | 0 | 16 | 0 | **5.3333** | no | no | Cell Wall | Rv2040c |
| Rv2041c | 8 | 0 | 16 | 0 | **5.3333** | no | no | Cell Wall | Rv2041c |
| Rv2437 | 8 | 0 | 16 | 0 | **5.3333** | no | no | Cell Wall | Rv2437 |
| Rv2617c | 8 | 0 | 16 | 0 | **5.3333** | no | no | Cell Wall | Rv2617c |
| Rv2620c | 8 | 0 | 16 | 0 | **5.3333** | no | no | Cell Wall | Rv2620c |
| Rv2625c | 8 | 0 | 16 | 0 | **5.3333** | no | no | Cell Wall | Rv2625c |
| Rv2686c | 8 | 0 | 16 | 0 | **5.3333** | no | no | Cell Wall | Rv2686c |
| Rv2687c | 8 | 0 | 16 | 0 | **5.3333** | no | no | Cell Wall | Rv2687c |
| Rv2688c | 8 | 0 | 16 | 0 | **5.3333** | no | no | Cell Wall | Rv2688c |
| Rv2693c | 8 | 0 | 16 | 0 | **5.3333** | no | no | Cell Wall | Rv2693c |
| Rv2729c | 8 | 0 | 16 | 0 | **5.3333** | no | no | Cell Wall | Rv2729c |
| Rv2877c | 8 | 0 | 16 | 0 | **5.3333** | no | no | Cell Wall | Rv2877c |
| Rv3197 | 8 | 0 | 16 | 0 | **5.3333** | no | no | Cell Wall | Rv3197 |
| Rv3289c | 8 | 0 | 16 | 0 | **5.3333** | no | no | Cell Wall | Rv3289c |
| Rv3335c | 8 | 0 | 16 | 0 | **5.3333** | no | no | Cell Wall | Rv3335c |
| Rv2093c | 8 | 0 | 16 | 0 | **5.3333** | no | no | Cell Wall | tatC |
| Rv2835c | 8 | 0 | 16 | 0 | **5.3333** | no | no | Cell Wall | ugpA |
| Rv2833c | 8 | 0 | 16 | 0 | **5.3333** | no | no | Cell Wall | ugpB |
| Rv2834c | 8 | 0 | 16 | 0 | **5.3333** | no | no | Cell Wall | ugpE |
| Rv2316 | 8 | 0 | 16 | 0 | **5.3333** | no | no | Cell Wall | uspA |
| Rv2780 | 0 | 4 | 8 | 0 | 4 | no | **yes** | Other | ald |
| Rv0062 | 0 | 7 | 0 | 0 | 2.3333 | no | **yes** | Other | celA1 |
| Rv2672 | 0 | 7 | 0 | 0 | 2.3333 | no | **yes** | Other | Rv2672 |
| Rv3883c | 0 | 0 | 0 | 0 | 0 | no | **yes** | Other | mycP1 |
| Rv3449 | 0 | 0 | 0 | 0 | 0 | no | **yes** | Other | mycP4 |
| Rv2957 | 0 | 0 | 0 | 0 | 0 | no | **yes** | Other | Rv2957 |
| Rv3668c | 0 | 0 | 0 | 0 | 0 | no | **yes** | Other | Rv3668c |
| Rv3671c | 0 | 0 | 0 | 0 | 0 | no | **yes** | Other | Rv3671c |
| **^1^**Genes are listed in the following order: 1) Genes of special interest: first known CD4 and CD8 antigens, then “ESAT-6 like” genes; 2) genes with function-adjusted weighting of ≥ 9 in descending order; 3) genes with a composite evidence-based average weight of ≥ 5.33; and 4) genes that were categorized as “secreted” in TubercuList but did not have a function-adjusted weighting ≥ 9.  **^2^**Bold font denotes the attribute used to include the gene in the library and determining the order of genes listed. | | | | | | | | | |
